# Long-term immunogenicity and immune memory response to the hepatitis B antigen in the RTS,S/AS01_E_ malaria vaccine in African children: a randomized trial

**DOI:** 10.1080/21645515.2019.1695457

**Published:** 2020-01-17

**Authors:** Innocent Valéa, Samuel Adjei, Effua Usuf, Ousmane Traore, Daniel Ansong, Halidou Tinto, Harry Owusu Boateng, Athanase Mwinessobaonfou Some, Patrick Buabeng, Johan Vekemans, Amos Kotey, Pascale Vandoolaeghe, Mark Cullinane, Magali Traskine, Florence Ouedraogo, David Sambian, Marc Lievens, Marc Christian Tahita, Erik Jongert, Palpouguini Lompo, Ali Idriss, Dorota Borys, Sayouba Ouedraogo, Frank Prempeh, Lode Schuerman, Hermann Sorgho, Tsiri Agbenyega

**Affiliations:** a Unité de Recherche Clinique de Nanoro, Institut de Recherche en Sciences de la Santé, Nanoro, Burkina Faso; b IRSS, Centre Muraz, Bobo-Dioulasso, Burkina Faso; c School of Medical Sciences, KNUST (Agogo Presbyterian Hospital), Kumasi, Ghana; d Vaccine, GSK, Wavre, Belgium

**Keywords:** Malaria, RTS, S/AS01_E_, vaccine, long-term immunogenicity, hepatitis B, safety, Expanded Program on Immunization

## Abstract

RTS,S/AS01_E_ malaria vaccine contains the hepatitis B virus surface antigen and may thus serve as a potential hepatitis B vaccine. To evaluate the impact of RTS,S/AS01_E_ when implemented in the Expanded Program of Immunization, infants 8–12 weeks old were randomized to receive either RTS,S/AS01_E_ or a licensed hepatitis B control vaccine (HepB), both co-administered with various combinations of the following childhood vaccines: diphtheria-tetanus-acellular pertussis-*Haemophilus influenzae* type b, trivalent oral poliovirus, pneumococcal non-typeable *Haemophilus influenzae* protein D conjugate and human rotavirus vaccine. Long-term persistence of antibodies against the circumsporozoite (CS) protein and hepatitis B surface antigen (HBsAg) were assessed, together with the immune memory response to the HB antigen following a booster dose of HepB vaccine. Subgroups receiving RTS,S or the HepB control vaccine were pooled into RTS,S groups and HepB groups, respectively. One month post-HepB booster vaccination, 100% of participants in the RTS,S groups and 98.3% in the control groups had anti-HBs antibody concentrations ≥10 mIU/mL with the geometric mean concentrations (GMCs) at 46634.7 mIU/mL (95% CI: 40561.3; 53617.6) and 9258.2 mIU/mL (95% CI: 6925.3; 12377.0), respectively. Forty-eight months post-primary vaccination anti-CS antibody GMCs ranged from 2.3 EU/mL to 2.7 EU/mL in the RTS,S groups compared to 1.1 EU/mL in the control groups. Hepatitis B priming with the RTS,S/AS01_E_ vaccine was effective and resulted in a memory response to HBsAg as shown by the robust booster response following an additional dose of HepB vaccine. RTS,S/AS01E when co-administered with PHiD-CV, HRV and other childhood vaccines, had an acceptable safety profile.

## Introduction

RTS,S/AS01_E_ is a *Plasmodium falciparum* malaria vaccine intended for routine immunization of infants and children in malaria-endemic regions in Sub-Saharan Africa as part of the Expanded Program on Immunization (EPI). The vaccine is designed to complement currently available measures to fight *P. falciparum* malaria and may therefore substantially contribute to existing malaria control programs. The RTS,S/AS01_E_ vaccine consists of the RTS,S ‘hybrid’ antigen; in which the central repeat region of the *Plasmodium falciparum* circumsporozoite (CS) protein, referred to as ‘R’, and the T-cell epitopes of the CS protein (‘T’) are fused to the hepatitis B virus surface antigen (HBsAg) referred to as ‘S’. The vaccine is formulated with the AS01_E_ Adjuvant System. RTS,S/AS01_E_ induces antibody responses to the CS protein and to HBsAg.^1^

The hepatitis B virus causes life-threating infections worldwide and poses a public health problem in Sub-Saharan Africa.^2^ Chronic hepatitis B is a risk factor for liver cirrhosis and liver cancer, and transmission of the virus occurs by exposure to the blood or body fluids of infected individuals.^2^ Given the health burden of chronic hepatitis B, vaccination in infants of Sub-Saharan African countries has become key to protect against the disease. RTS,S/AS01_E_ contains the HBsAg and may thus serve as an additional hepatitis B vaccine dose. In line with the recommendation to vaccinate all infants against hepatitis B virus,^3^ RTS,S/AS01_E_ was co-administered in recent studies with diphtheria-tetanus-whole cell pertussis (DTPw)-based pentavalent vaccines, which contains the hepatitis B surface antigen.^4,5^ The data from these studies show that co-administration of RTS,S/AS01_E_ with licensed vaccines containing the hepatitis B surface antigen has an acceptable safety profile and no deleterious effect on anti-hepatitis B virus immune response.

Based on the positive benefit-risk balance of RTS,S/AS01_E_ and its potential for substantial impact against both clinical and severe malaria, the European Medicines Agency (EMA) provided a positive scientific opinion for the RTS,S vaccine in children aged 6 weeks to 17 months (at the time of the first dose) in 2015.^6^ In 2016, the World Health Organization (WHO) recommended pilot implementation of the vaccine in children as of 5 months of age in 3 to 5 moderate-to-high malaria transmission settings in Sub-Saharan Africa.^7^

Primary study results, published in Valéa *et al*.^8^ support the indication of the RTS,S/AS01_E_ vaccine for immunization of infants against hepatitis B in settings where prevention of *P. falciparum* malaria is also being sought. These results demonstrated that RTS,S/AS01_E_ was non-inferior to a licensed HepB vaccine in terms of anti-HBs seroprotection rates at one month post-primary vaccination. Immune responses to PHiD-CV co-administered with RTS,S/AS01_E_ were non-inferior to PHiD-CV co-administered with HepB for 9 out of 10 vaccine serotypes (all except serotype 18C). Immune responses to HRV co-administered with RTS,S/AS01_E_ were non-inferior to HRV co-administered with HepB. RTS,S/AS01_E_ when co-administered with PHiD-CV, HRV and other vaccines included in the EPI had an acceptable safety profile during the 26 month follow-up period.^8^

As part of the long-term follow-up of this phase III study, we assessed long-term antibody persistence and safety of the RTS,S/AS01_E_ vaccine (up to 48 months post-primary vaccination series), the presence of anti-HBs immune memory and the anti-HBs immune response to HepB booster vaccination given 4 years after primary vaccination.

## Methodology

### Study design and participants

This phase III, open, randomized study (NCT01345240) was designed to compare the anti-HBs immune response induced by primary vaccination with RTS,S/AS01_E_ to that induced by primary vaccination with a licensed hepatitis B virus vaccine (HepB; *Engerix B, GSK*). The study was conducted at the Clinical Research Unit of Nanoro in Burkina Faso, and the Malaria Research Center Agogo Presbyterian Hospital/Kwame Nkrumah University of Science and Technology in Ghana. Infants 8–12 weeks old were randomized to receive either RTS,S/AS01_E_ or a licensed HepB control, both co-administered (concomitantly or staggered) with the following vaccines; diphtheria-tetanus-acellular pertussis-*Haemophilus influenzae* type b (DTaP/Hib, *Infanrix* Hib, GSK), trivalent oral poliovirus vaccine (tOPV, *Polio Sabin, GSK*), pneumococcal non-typeable *Haemophilus influenzae* protein D conjugate vaccine (PHiD-CV, *Synflorix, GSK*) and human rotavirus vaccine (HRV, *Rotarix, GSK*).^8^ The follow-up study was conducted at both study sites in Burkina Faso and Ghana between November 2011 and February 2017. All children received a HepB booster vaccination 48 months post-primary vaccination. The HepB vaccine contained 10 µg of purified hepatitis B surface antigen adsorbed onto aluminum hydroxide containing 0.25 mg of aluminum. RTS,S consists of the central R repeat region and the c-term T cell epitopes of the CS-protein (named “RT”) and hepatitis B surface antigen (named “S”). Per molecule of RT, there are 5 molecules of S (of which 1 S in fusion with RT and 4 “free” S). Molecular weights are quite similar for RT and S, which means that in a 25 µg RTS,S dose there is approximately 4.2 µg RT and 20.8 µg S. AS01 is a liposome-based adjuvant comprising 3-O-desacyl-4′-monophosphoryl lipid A (MPL – produced by GSK), a Toll-like receptor 4 ligand and QS-21, a saponin extracted from the bark of the *Quillaja saponaria* Molina tree (Licensed by GSK from Antigenics LLC, a wholly owned subsidiary of Agenus Inc., a Delaware, USA corporation).

Immunogenicity and safety data were collected up to one month after HepB booster vaccination. Details about the co-administered vaccines can be found in the Table 1. Data for participants receiving the different RTS,S/AS01_E_ (RTS,S groups) or HepB (control groups) primary vaccination regimens (both co-administered in various combinations with different study vaccines),^8^ were pooled in RTS,S groups and control groups for the analysis and comparison of HepB antibody persistence and booster response.

**Table 1.** Demographic characteristics per primary vaccination with RTS,S/AS01_E_ or HepB and co-administered vaccines (ATP cohort for immunogenicity – FU2).

Characteristics	Parameters	Group R1 (N = 131)	Group R2 (N = 111)	Group R3 (N = 122)	Group C1 (N = 127)	Group C2 (N = 107)
Co-administered vaccines		RTS,S DTaP/Hib tOPV PHiD-CV	RTS,S DTaP/Hib tOPV HRV	RTS,S DTaP/Hib tOPV	HepB DTaP/Hib tOPV PHiD-CV	HepB DTaP/Hib tOPV HRV
Staggered vaccines (2 weeks later)		HRV	PHiD-CV	PHiD-CV HRV	HRV	PHiD-CV
Age at HepB booster vaccination (years)	Mean (SD)	4.3 (0.0)	4.3 (0.0)	4.3 (0.0)	4.3 (0.0)	4.3 (0.0)
Gender	Male, n (%)	81 (61.8)	53 (47.7)	68 (55.7)	55 (43.3)	61 (57.0)
	Female, n (%)	50 (38.2)	58 (52.3)	54 (44.3)	72 (56.7)	46 (43.0)
		RTS,S (N = 364)			Control (N = 234)	
Age at HepB booster vaccination (years)	Mean (SD)	4.3 (0.0)			4.3 (0.0)	
Gender	Male, n (%)	202 (55.5)			116 (49.6)	
	Female, n (%)	162 (44.5)			118 (50.4)	

ATP, according-to-protocol; DTaP/Hib, diphtheria-tetanus-acellular pertussis- *Haemophilus influenzae* type b; FU2, follow-up 2; HepB, hepatitis B vaccine; HRV, human rotavirus vaccine; RTS,S/AS01_E_, malaria vaccine; SD, standard deviation; N, total number of participants; n (%), number (percentage) of participants in the specified category. PHiD-CV, pneumococcal non-typeable *Haemophilus influenzae* protein D conjugate vaccine; tOPV, trivalent oral poliovirus vaccine;

RTS,S, all study groups who received primary vaccination with RTS,S/AS01_E_; control, all study groups who received primary vaccination with HepB.

During the follow-up period, we collected blood samples and assessed the persistence of the immune response against HBsAg at 12, 24, 36 and 48 months post-dose 3. To evaluate the memory response to HBs antigen, HBs antibody titers were measured one-month after administration of the HepB booster dose. Anti-CS antibodies persistence was also measured at 12, 36 and 48 months post-dose 3. More information about the study design and the study vaccines can be found in Valéa *et al*.^8^

We performed the study in accordance with the principles of Good Clinical Practice, the Declaration of Helsinki and all applicable regulatory requirements. The concerned ethics committees and regulatory authorities reviewed and approved the study protocol, including amendments, and the informed consent form. Parents or legal guardians of each child gave written informed consent. The study is registered at www.clinicaltrials.gov (NCT01345240) and a protocol summary is available at http://www.gsk-clinicalstudyregister.com (GSK study number 113681).

### Objectives

The immunogenicity objectives during this follow-up study included the assessment of long-term antibody persistence following previous vaccination with RTS,S/AS01_E_ in terms of anti-HBs antibodies compared to a primary vaccination regimen with HepB, and of anti-CS antibodies. Other immunogenicity objectives were to study the antibody responses to HBsAg induced by the booster dose of the HepB vaccine. The safety objective included collection of serious adverse events (SAEs) and potential immune-mediated disorders (pIMDs) up to study end.

### Immunogenicity assessment

We measured antibodies against HBs using a chemiluminescence enzyme immunoassay (Centaur, Siemens Healthcare) with a cutoff of 6.2 mIU/mL at GSK Biologicals (Rixensart, Belgium). An anti-HBs concentration ≥10 mIU/mL is considered seroprotective.^9^ An additional analysis was done using a threshold of ≥ 100 mIU/mL for consistency with other clinical studies and given the potential for low anti-HBs levels to mask significant infection.^10^ Considering that antibody responses to the RF1 epitope of the HepB surface antigen are indicative of the virus-neutralizing capacity of the humoral immune response,^11,12^ we quantified RF1-like antibody levels using an in-house developed enzyme-linked immunosorbent assay (ELISA) based competition assay with plate adsorbed HBsAg at the CEVAC laboratory (University of Gent, Gent, Belgium). The assay cutoff was 33 EU/mL. We determined antibodies against CS by ELISA at the CEVAC laboratory (University of Gent, Gent, Belgium). Due to revalidation with new assay agents, the cutoff for the CS assay was changed from 0.5 EU/mL used for month 1 and month 12 post dose 3 to 1.9 EU/mL applied for month 36 and month 48 post-dose 3.

### Safety assessment

All solicited adverse events (AEs) were reported during the 7-day period following each vaccine dose and all unsolicited AEs during the 30-day period following each vaccine dose. All SAEs with causal relationship to vaccination, fatal SAEs and AE of special interest (AESI) including potential pIMDs from study week 0 until one-month post-booster HepB vaccination were described.

### Statistical analyses

We based the immunogenicity analysis on the according-to-protocol (ATP) cohort for immunogenicity analysis of follow-up 1 (FU1, months 12 and 24 post-primary vaccination) and follow-up 2 (FU2, months 36 and 48 post-primary vaccination and one month post-booster HepB vaccination). The ATP cohort for immunogenicity analysis FU1 and FU2 contained all evaluable participants from the ATP cohort for analysis of immunogenicity excluding participants who did not follow inclusion criteria of immunogenicity during the first and second follow-up time, respectively (see Supplementary material). Immunogenicity analysis was performed by pooled groups for the anti-HBs immune response. The total vaccinated cohort (TVC) included all participants with at least one vaccine administration documented. We performed the safety analysis on the TVC per treatment actually administered. The geometric mean concentration (GMCs) calculations were performed by taking the anti-log of the mean of the log concentration transformations. Antibody titers/concentrations below the cutoff of the assay were given an arbitrary value of half the cutoff for the purpose of GMC calculation.

## Results

### Demographic characteristics

**Figure 1.**
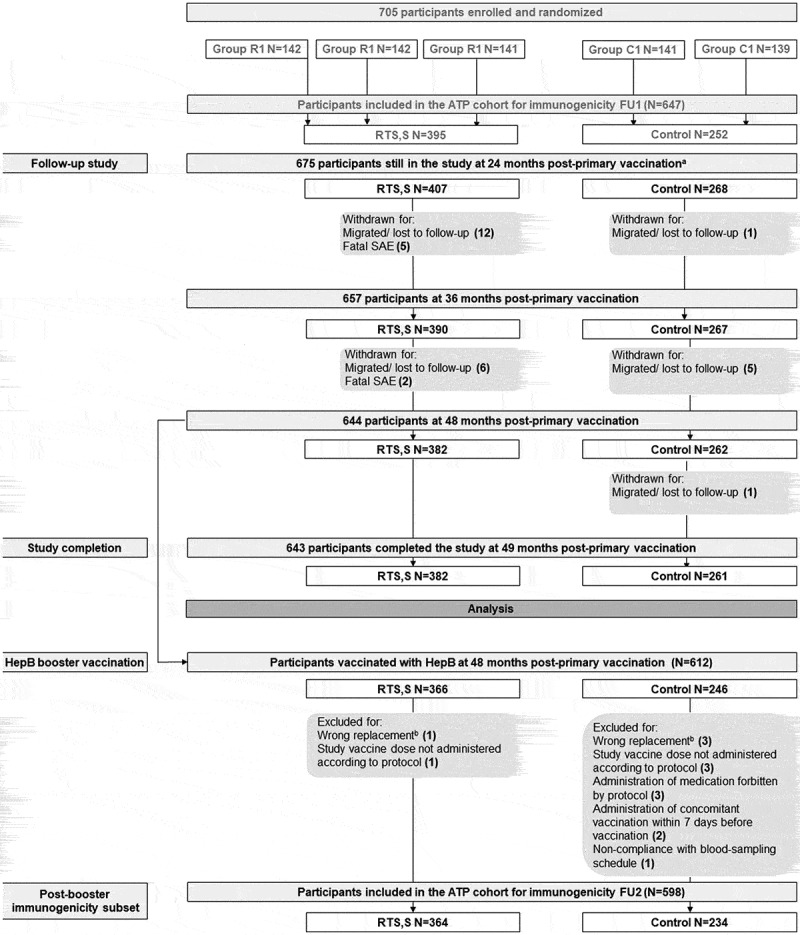
Participants flowchart.

As previously presented in *Valea et al*.^8^ 705 participants (425 in pooled RTS,S groups and 280 in pooled control groups) were enrolled in the primary study and randomized. Among them, 675 completed the visit 24 months post-primary vaccination. Six hundred and forty-three participants completed the study at one month post-booster HepB (Figure 1). Forty-eight months post-primary vaccination, 612 participants were vaccinated and 598 were included in the ATP cohort for immunogenicity FU2 (for more details see Figure 1). Thirty-two participants (25 in the RTS,S groups and 7 in the control groups) withdrew during FU2.The mean age at HepB booster vaccination was 4.3 years. Gender distribution was balanced between all groups (Table 1).

### Anti-CS immune response

#### Persistence of anti-CS antibodies

The seropositivity rates and GMCs for anti-CS antibodies one month following primary vaccination were already described in Valea *et al*., 2018.^8^ Forty-eight months post-primary vaccination, seropositivity rates ranged in the RTS,S groups from 56.3% to 59.8%, with anti-CS antibody GMCs ranging from 2.3 EU/mL to 2.7 EU/mL compared to 1.1 EU/mL in the control groups (Supplementary Table 1).

### Anti-HBs immune response

#### Persistence of anti-HBs antibodies

The percentage of participants with anti-HBs antibody concentrations ≥10 mIU/mL remained high between month 1 and month 48 post-dose 3 in the RTS,S groups (month 1, 100%; month 48, 99.4%), and was lower in the control groups (month 1, 96.0%; month 48, 73.8%) (Table 2). The percentage of participants with anti-HBs antibody concentrations ≥100 mIU/mL was lower at month 48 when compared to month 1 for participants in both the RTS,S and control groups (100% one month post-dose 3 and 92.0% at month 48 in the RTS,S groups; 83.4% at month 1 to 24.0% at month 48 in the control groups) (Table 2).

**Table 2.** Anti-HBs seroprotection rates and antibody GMCs following HepB booster vaccination at 4 years post-primary in participants who previously received primary vaccination with RTS,S/AS01_E_ or HepB (ATP cohort for immunogenicity).

Group	Timing	N	% (95% CI) ≥ 6.2 mIU/mL	% (95% CI) ≥ 10 mIU/mL	% (95% CI) ≥ 100 mIU/mL	GMC, mIU/mL (95% CI)
RTS,S	Pre-vaccination	398	20.9 (17.0; 25.2)	15.8 (12.4; 19.8)	4.3 (2.5; 6.8)	5.0 (4.5; 5.7)
	1 month post-dose 3	397	100 (99.1; 100)	100 (99.1; 100)	100 (99.1; 100)	6412.7 (5732.9; 7173.0)
	12 months post-dose 3	380	100 (99.0; 100)	100 (99.0; 100)	98.9 (97.3; 99.7)	1995.0 (1784.5; 2230.5)
	24 months post-dose 3	365	100 (99.0; 100)	100 (99.0; 100)	96.4 (94.0; 98.1)	1540.4 (1345.1; 1764.1)
	36 months post-dose 3	355	99.7 (98.4; 100)	99.4 (98.0; 99.9)	94.9 (92.1; 97.0)	896.6 (780.2;1030.3)
	48 months post-dose 3	361	99.4 (98.0; 99.9)	99.4 (98.0; 99.9)	92.0 (88.7; 94.6)	669.8 (579.7; 773.9)
	1 month post-booster HepB	362	100 (99.0; 100)	100 (99.0; 100)	99.4 (98.0; 99.9)	46634.7 (40561.3; 53617.6)
Control	Pre-vaccination	251	22.7 (17.7; 28.4)	17.9 (13.4; 23.2)	5.6 (3.1; 9.2)	5.4 (4.7; 6.4)
	1 month post-dose 3	253	97.2 (94.4; 98.9)	96.0 (92.9; 98.1)	83.4 (78.2; 87.8)	377.4 (310.6; 458.7)
	12 months post-dose 3	241	94.6 (91.0; 97.1)	92.9 (88.9; 95.8)	63.5 (57.1; 69.6)	127.7 (105.0; 155.4)
	24 months post-dose 3	235	88.5 (83.7; 92.3)	83.8 (78.5; 88.3)	46.4 (39.9; 53.0)	69.8 (56.1; 86.9)
	36 months post-dose 3	230	83.0 (77.6; 87.7)	77.4 (71.4; 82.6)	31.3 (25.4; 37.7)	40.0 (32.2; 49.7)
	48 months post-dose 3	233	79.4 (73.6; 84.4)	73.8 (67.7; 79.3)	24.0 (18.7; 30.0)	30.8 (24.9; 38.1)
	1 month post-booster HepB	229	98.3 (95.6; 99.5)	98.3 (95.6; 99.5)	95.6 (92.1; 97.9)	9258.2 (6925.3; 12377.0)

ATP, according-to-protocol; CI, confidence interval; FU, follow-up; GMC, geometric mean antibody concentration; HepB, hepatitis B vaccine; IU, international unit; M, month; N, total number of participants; %, percentage of participants with antibody concentrations above the specified cutoff; RTS,S/AS01_E_, malaria vaccine. RTS,S groups, all study groups who received primary vaccination with RTS,S/AS01_E_; control, all study groups who received primary vaccination with HepB; post-dose 3, blood sampling after the third dose, x months after primary vaccination course; post-booster HepB, blood sampling 1 month after HepB booster dose.

Between month 1 and month 48 post-dose 3, anti-HBs antibody GMCs declined sharply in both the RTS,S groups (from 6412.7 mIU/mL to 669.8 mIU/mL) and the control groups (from 377.4 mIU/mL to 30.8 mIU/mL) (Table 2). Forty-eight months post-primary vaccination, anti-HBs antibody GMC was 22-fold higher in the RTS,S groups than in the control groups.

### Anti-HBs antibody responses following HepB booster vaccination

One month post HepB booster vaccination, 100% of participants in the RTS,S groups, and 98.3% in the control groups had antibody titers ≥10 mIU/mL; 99.4% in the RTS,S group, and 95.6% in the control groups had antibody titers ≥100 mIU/mL (Table 2). Anti-HBs antibody GMCs were 46634.7 mIU/mL and 9258.2 mIU/mL one month post-HepB booster vaccination in the RTS,S and control groups, respectively. Compared to pre-booster values, anti-HBs antibody GMCs increased 70-fold in the RTS,S groups and 300-fold in the control groups (Table 2).

### Antibody responses HBs RF1-like antigens

One month post-primary vaccination, 97.5% of participants in the RTS,S groups had anti-HBs RF1 antibody titers ≥33 EU/mL compared to 35.3% in the control groups. One month post-HepB booster vaccination, 95% of participants had antibody concentrations above this cutoff in the RTS,S groups compared to 72.8% in the control groups (Table 3). Anti-HBs RF1 antibody GMCs were 306.9 EU/mL and 27.1 EU/mL one month post-primary vaccination and 415.7 EU/mL and 123.9 EU/mL one month post-HepB booster vaccination in the RTS,S and control groups, respectively (Table 3).

**Table 3.** Seropositivity rate and GMCs for anti-HBs RF1-like antibodies in participants who received primary vaccination with RTS,S/AS01_E_ versus HepB, at 1 month after primary vaccination and 1 month after HepB booster dose (ATP cohort for immunogenicity-FU2).

Group	Timing	N	% (95% CI) ≥ 33 EU/mL	GMC, EU/mL (95% CI)
RTS,S	1 month post-dose 3	362	97.5 (95.3; 98.9)	306.9 (276.0; 341.3)
	1 month post-booster HepB	341	95.0 (92.1; 97.1)	415.7 (362.2; 476.9)
Control	1 month post-dose 3	232	35.3 (29.2; 41.9)	27.1 (24.5; 29.9)
	1 month post-booster HepB	202	72.8 (66.1; 78.8)	123.9 (99.1; 155.0)

ATP, according-to-protocol; CI, confidence interval; EU, enzyme-linked immunosorbent assay unit; FU2, follow-up 2; GMC, geometric mean concentration; HepB, hepatitis B vaccine; anti-HBs, antibodies to the hepatitis B surface antigen; M, month; N, total number of participants; %, percentage of participants with antibody concentration equal to or above the specified cut off. RTS,S, all study groups who received primary vaccination with RTS,S/AS01_E_; control, all study groups who received primary vaccination with HepB; post-dose 3, blood sampling one month after primary vaccination course.

### Safety

No SAEs considered causally related to study vaccination and no pIMDs were reported during the entire study period. In the first 24 months of follow-up, 5/425 (1.2%) fatal SAEs occurred in the RTS,S, versus 3/280 (1.1%) in the control groups (age of onset is between 2 and 26 months). Over approximately 4 years between study start and one month post- HepB booster vaccination, 12/425 (2.8%) fatal SAEs occurred in the RTS,S groups (5 in girls and 7 in boys) and 3/280 (1.1%) in the control groups (all in girls) (Supplementary Table 2). No pattern/explanation for this difference could be identified and most of the deaths were due to various infectious diseases (Supplementary Table 2).

## Discussion

The present follow-up study indicated a general steady decline in anti-CS antibody levels over the 49-month follow-up period. This observation indicates that no anti-CS boosting was occurring following natural exposure to malaria.

We previously demonstrated that the immune response against HBsAg induced by 3-dose priming with RTS,S/AS01_E_ was non-inferior to 3 dose priming with a licensed HepB vaccine.^8^ This study showed that anti-HBs antibody GMCs declined from one month post-primary vaccination to 48 months post-primary vaccination in all groups. Antibody GMCs remained however 22-fold higher 48 months post-primary vaccination in participants who received the RTS,S/AS01_E_ vaccine compared to participants who received the licensed HepB control vaccine, as expected from the higher HepB dose antigens and the adjuvant (AS01_E_) provided with the RTS,S/AS01_E_ vaccine.

A HepB booster dose 48 months post-primary vaccination induced a robust immune response in participants who received 3 priming doses of either RTS,S/AS01_E_, or a licensed HepB vaccine. Consistent with previous experience in children who received HepB vaccine, HBs antibody titers may wane to low or undetectable concentrations 5–15 years following primary vaccination,^13^ and persistence is dependent on peak anti-HBs levels.^14^ Nevertheless, immune responses to booster vaccination remained robust, even if levels drop below 10 mIU/mL, as previously demonstrated.^14^ In the present study, the booster response was robust irrespective of the primary vaccination schedule, suggesting that all participants mounted immune memory to HBsAg.

Detectable anti-HBs antibody titers pre-primary vaccination in about 20% of participants may represent maternal transfer of antibodies. We believe that interpretation of the results was not affected by these findings as to our knowledge, maternal antibodies may have very little impact on a booster response to an additional HepB dose 48 months after primary vaccination. Additionally, prevalence of children with detectable anti-HBs antibody titers seemed balanced between RTS,S groups.

To assess the virus-neutralizing capacity of the humoral immune response,^11,12^ antibody responses to the RF1 epitope of the HBsAg were assessed and were higher in the RTS,S groups compared to the control groups, as shown by the seropositivity rates and GMCs one month after both primary and booster vaccination. This is consistent with previous results in infants 6–12 weeks of age at first vaccination, at one month post-primary vaccination with 3 doses of RTS,S/AS01_E_, according to a 0,1,2-month schedule.^3,4^ In the current study, higher anti-HBs RF1-like antibody GMCs were observed in both groups following booster vaccination compared to one month post-primary vaccination, indicative of a booster response. A correlated of protection exists for HepB immunization. Whether the higher anti-HBs RF1-like antibody GMCs following booster vaccination in the RTS,S groups compared to the control groups will result in a clinically meaningful benefit in terms of longer-term protection remains unknown and the clinical benefit may be limited.

Overall, RTS,S/AS01_E_ was well tolerated. While the numbers of deaths were similar between groups up to 24 months post-primary vaccination (1.2% (5) versus 1.1% (3) in RTS,S and control groups, respectively), differences emerged later on during the study period (1.9% (7) versus 0 in the RTS,S and control groups, respectively). Among these 7 fatal SAEs reported after M24, 2 bacterial meningitis cases and 2 severe malaria cases were reported (Supplementary Table 2). There were no SAEs, including SAEs with a fatal outcome, that were considered causally-related to RTS,S/AS01_E_ and no pIMDs reported during the entire study follow-up period. More data on meningitis cases, severe malaria and mortality overall and by gender will be coming from the ongoing large-scale pilot implementations of the vaccine in Malawi, Ghana and Kenya, as recommended by the WHO. No gender-specific mortality imbalance was observed within the RTS,S groups.

Follow-up of a large pool of children for 4–5 years is difficult to achieve and presents a clear strength of this study. A limitation of the study is the open label design. Overall, there were no non-responders and seroprotection against HepB was achieved in participants irrespective of the treatment group they were allocated to.

**Figure 2.**
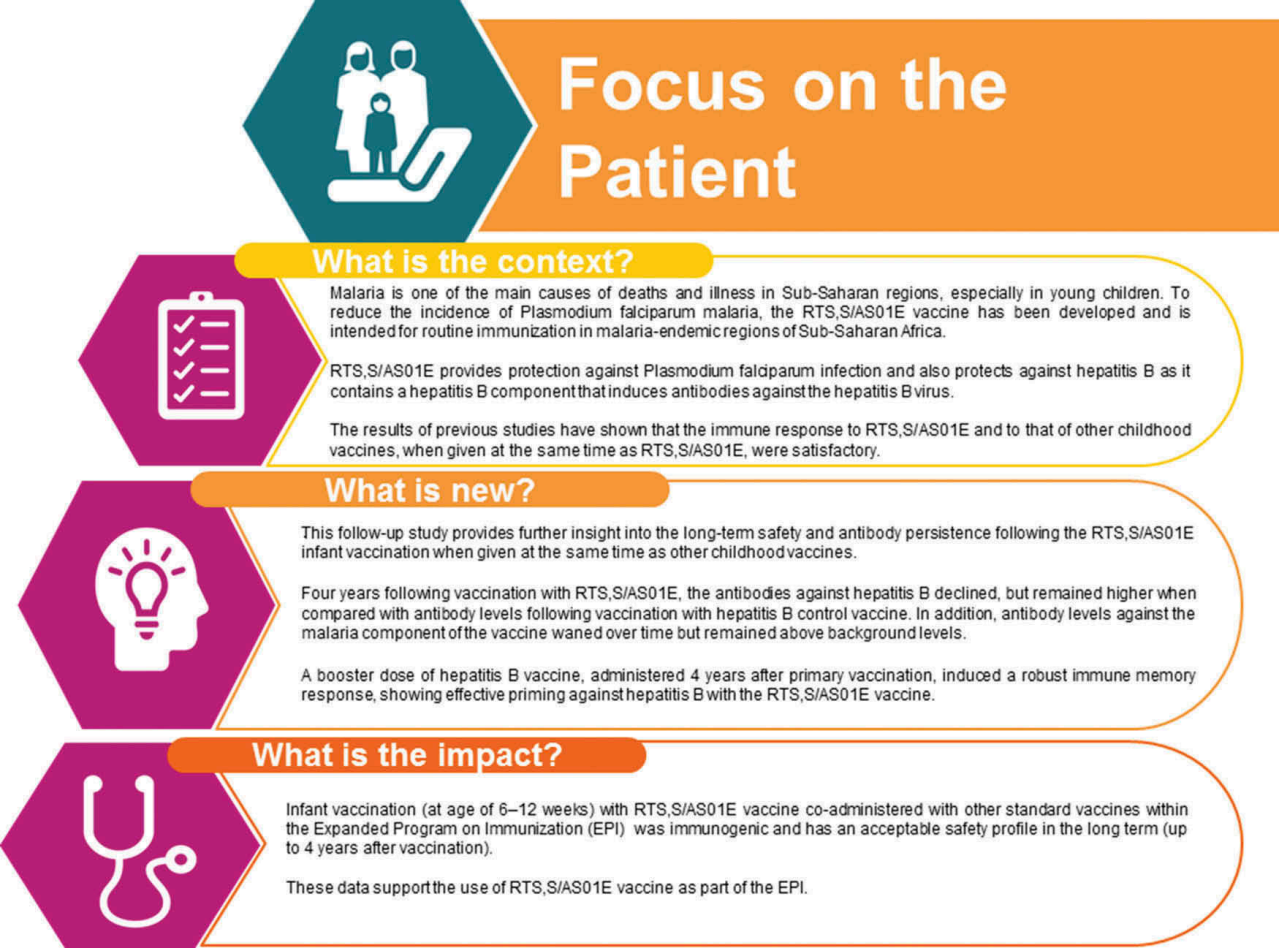
Focus on patient section.

Figure 2 represents a Focus on Patient Section, which elaborates on the research clinical relevance that could be shared to patients by Health Care Professionals.

## Conclusions

Anti-HBs priming with the RTS,S/AS01E malaria vaccine is effective and results in a memory response to HBsAg as shown by the booster response leading to anti-HBs antibody GMCs after the additional dose of HepB vaccine 4 years post-priming at least as high as the licensed HepB vaccine.

## Supplementary Material

Supplemental Material

## Abbreviations

CS: circumsporozoite
DTaP/Hib: diphtheria-tetanus-acellular pertussis- *Haemophilus influenza*e type b
EPI: Expanded Program on Immunization
EU: enzyme-linked immunosorbent assay unit
FU1(2): follow-up 1(2)
GMC: geometric mean concentration
anti-HBs: antibodies to the hepatitis B surface antigen
HBsAg: hepatitis B virus surface antigen
HepB: hepatitis B
HRV: human rotavirus vaccine
IU: international unit
PHiD-CV: pneumococcal non-typeable *Haemophilus influenzae* protein D conjugate vaccine
PIMDs: potential immune-mediated disorders
RF1 epitope: an immunogenic epitope of hepatitis B virus recognized by neutralizing antibody RF1
SAE: serious adverse event
tOPV: trivalent oral poliovirus vaccine
TVC: total vaccinated cohort.

## Data Sharing Statement

Anonymized individual participant data and study documents can be requested for further research from www.clinicalstudydatarequest.com.

## Trademark statement

*Engerix-B, Rotarix, Synflorix, Infanrix* and *Polio Sabin* are trademarks owned by, or licensed to, the GSK group of companies.

## References

[1] European Medicines Agency. Committee for Medicinal Products for Human Use (CHMP). Assessment Report: Mosquirix^TM^. [Accessed 2018 115]. http://bit.ly/2paE1uw.

[2] World Health Organization. Hepatitis B. Key facts; 7 2019 [Accessed 2019 712]. https://www.who.int/en/news-room/fact-sheets/detail/hepatitis-b.

[3] World Health Organization. WHO position paper on hepatitis B vaccines. Weekly epidemiological record No. 40. 2009; 84:405–20. [Accessed 4 2019]. https://www.who.int/wer/2009/wer8440.pdf?ua=1.

[4] Asante KP, Abdulla S, Agnandji S, Lyimo J, Vekemans J, Soulanoudjingar S, Owusu R, Shomari M, Leach A, Jongert E, et al. Safety and efficacy of the RTS,S/AS01E candidate malaria vaccine given with expanded-programme-on-immunisation vaccines: 19 month follow-up of a randomised, open-label, phase 2 trial. Lancet Infect Dis. 2011;11(10):741–49. doi:10.1016/S1473-3099(11)70100-1. 21782519

[5] RTSS Clinical Trials Partnership. A phase 3 trial of RTS,S/AS01 malaria vaccine in African infants. N Engl J Med. 2012;367(24):2284–95. doi:10.1056/NEJMoa1208394. 23136909 PMC10915853

[6] European Medicines Agency. Committee for Medicinal Products for Human Use (CHMP) summary of opinion, mosquirix. [Accessed 511 2018]. http://www.ema.europa.eu/docs/en_GB/document_library/Medicine_for_use_outside_EU/2015/07/WC500190452.pdf.

[7] World Health Organization. Malaria vaccine: WHO position paper-January 2016. Wkly Epidemiol Rec. 2016;91(4):33–51. 26829826

[8] Valea I, Adjei S, Usuf E, Traore O, Ansong D, Tinto H, Owusu Boateng H, Leach A, Mwinessobaonfou Some A, Buabeng P, et al. Immune response to the hepatitis B antigen in the RTS,S/AS01 malaria vaccine, and co-administration with pneumococcal conjugate and rotavirus vaccines in African children: A randomized controlled trial. Hum Vaccin Immunother. 2018;14(6):1489–500. doi:10.1080/21645515.2018.1442996. 29630438 PMC6037440

[9] World Health Organization. Immunization, vaccines and biologicals.The immunological basis for immunization series. Module 22: Hepatitis B. [Accessed 2018 115]. http://apps.who.int/iris/bitstream/handle/10665/77755/9789241504751_eng.pdf?sequence=1.

[10] Gerlich WH. The enigma of concurrent hepatitis B surface antigen (HBsAg) and antibodies to HBsAg. Clin Infect Dis. 2007;44(9):1170–72. doi:10.1086/513296. 17407034

[11] Iwarson S, Tabor E, Thomas HC, Goodall A, Waters J, Snoy P, Shih JW, Gerety RJ. Neutralization of hepatitis B virus infectivity by a murine monoclonal antibody: an experimental study in the chimpanzee. J Med Virol. 1985;16(1):89–96. doi:10.1002/(ISSN)1096-9071. 2413167

[12] Waters JA, Pignatelli M, Brown D, O’Rourke S, Lever A, Thomas HC. The immune response to hepatitis B virus. Postgrad Med J. 1987;63:51–56. 2446303

[13] Leuridan E, Van Damme P. B and the need for a booster dose. Clin Infect Dis. 2011;53(1):68–75. doi:10.1093/cid/cir270. 21653306

[14] Jilg W, Schmidt M, Deinhardt F. Four-year experience with a recombinant hepatitis B vaccine. Infection. 1989;17(2):70–76. doi:10.1007/BF01646879. 2714860

