# Supplementary material for: Long-term immunogenicity and immune memory response to the hepatitis B antigen in the RTS,S/AS01E malaria vaccine in African children: a randomized trial
Source: Hum Vaccin Immunother. 2020 Jan 17;16(6):1464–70. doi: 10.1080/21645515.2019.1695457 (PMC7482624; doi:10.1080/21645515.2019.1695457)
Supplement: Supplemental Material [file KHVI_A_1695457_SM1032.zip › Supplementary table 1.docx]

Supplementary Table 1. Anti-CS seropositivity rates and antibody GMCs per co-administration primary vaccination regimen 1, 12, 36 and 48 months after primary vaccination (ATP cohort for immunogenicity-FU2)

| **Group** | **Timing** | **N** | **Seropositivity**  **% (95% CI),**  **≥ 0.5 EU/mL^1^ or ≥ 1.9 EU/mL^2^** | **GMC,**  **EU/mL (95% CI)** |
| --- | --- | --- | --- | --- |
| Group R1 | 1 month post-dose 3 | 141 | 100 (97.4; 100) | 142.2 (116.4; 173.7) |
|  | 12 months post-dose 3 | 91 | 91.2 (83.4; 96.1) | 5.7 (4.2; 7.7) |
|  | 36 months post-dose 3 | 131 | 55.7 (46.8; 64.4) | 2.6 (2.2; 3.1) |
|  | 48 months post-dose 3 | 128 | 56.3 (47.2; 65.0) | 2.3 (2.0; 2.7) |
| Group R2 | 1 month post-dose 3 | 123 | 100 (97.0; 100) | 188.5 (156.5; 227.0) |
|  | 12 months post-dose 3 | 82 | 95.1 (88.0; 98.7) | 6.8 (5.0; 9.4) |
|  | 36 months post-dose 3 | 107 | 61.7 (51.8; 70.9) | 2.8 (2.3; 3.4) |
|  | 48 months post-dose 3 | 111 | 57.7 (47.9; 67.0) | 2.4 (2.0; 2.8) |
| Group R3 | 1 month post-dose 3 | 136 | 99.3 (96.0; 100) | 205.5 (167.3; 252.5) |
|  | 12 months post-dose 3 | 96 | 90.6 (82.9; 95.6) | 7.5 (5.3; 10.6) |
|  | 36 months post-dose 3 | 121 | 70.2 (61.3; 78.2) | 3.5 (2.9; 4.2) |
|  | 48 months post-dose 3 | 122 | 59.8 (50.6; 68.6) | 2.7 (2.3; 3.2) |
| Group C1 | 1 month post-dose 3 | 135 | 11.9 (6.9; 18.5) | 0.3 (0.3; 0.3) |
|  | 12 months post-dose 3 | 85 | 10.6 (5.0; 19.2) | 0.3 (0.3; 03.) |
|  | 36 months post-dose 3 | 126 | 6.3 (2.8; 12.1) | 1.0 (1.0; 1.1) |
|  | 48 months post-dose 3 | 127 | 10.2 (5.6; 16.9) | 1.1 (1.0; 1.1) |
| Group C2 | 1 month post-dose 3 | 118 | 10.2 (5.4; 17.1) | 0.3 (0.3; 0.4) |
|  | 12 months post-dose 3 | 76 | 9.2 (3.8; 18.1) | 0.3 (0.3; 0.4) |
|  | 36 months post-dose 3 | 106 | 4.7 (1.5; 10.7) | 1.0 (1.0; 1.0) |
|  | 48 months post-dose 3 | 107 | 11.2 (5.9; 18.8) | 1.1 (1.0; 1.3) |

Group R1 received RTS,S/AS01_E_ + (DTaP/Hib + tOPV + PHiD-CV), and HRV 2 weeks later, Group R2 received RTS,S/AS01_E_ + (DTaP/Hib + tOPV + HRV), and PHiD-CV 2 weeks later, Group R3 received RTS,S/AS01_E_ + (DTaP/Hib + tOPV), and (PHiD-CV + HRV) 2 weeks later, Group C1 received HepB + (DTaP/Hib + tOPV + PHiD-CV), and HRV 2 weeks later, Group C2 received HepB + (DTaP/Hib + tOPV + HRV), and PHiD-CV 2 weeks later.

ATP, according-to-protocol; DTaP/Hib, diphtheria-tetanus-acellular pertussis- *Haemophilus influenzae* type b; EU, enzyme-linked immunosorbent assay unit; FU2, follow-up 2; GMC, geometric mean antibody concentration calculated on all participants; HepB, hepatitis B vaccine; HRV, human rotavirus vaccine; M, month; N, number of participants with available results; RTS,S/AS01_E_, malaria vaccine; PHiD-CV, pneumococcal non-typeable *Haemophilus influenzae* protein D conjugate vaccine; tOPV, trivalent oral poliovirus vaccine; %, percentage of participants with concentration equal to or above the cut-off.

^1^ seropositivity cut-off: ≥ 0.5 EU/mL was used at 1 month and 12 months post-dose 3, ^2^ seropositivity cut-off: ≥ 1.9 EU/mL was used at 36 and 48 months post-dose 3; 95% CI, 95% confidence interval; post-dose 3, blood sampling after the third dose, x months after primary vaccination course.
