# Supplementary material for: Long-term immunogenicity and immune memory response to the hepatitis B antigen in the RTS,S/AS01E malaria vaccine in African children: a randomized trial
Source: Hum Vaccin Immunother. 2020 Jan 17;16(6):1464–70. doi: 10.1080/21645515.2019.1695457 (PMC7482624; doi:10.1080/21645515.2019.1695457)
Supplement: Supplemental Material [file KHVI_A_1695457_SM1032.zip › Supplementary Table 2.docx]

**Supplementary Table 2: Fatal Serious Adverse Events reported from study start until 1 month after HepB booster dose (Total vaccinated cohort)**

| Group | Participant arbitrary number | Sex | Country | Age at onset (month) | Verbatim | Preferred term | Primary system organ class | MED type | Dose | Day of onset | Duration (days) | Intensity | Causality |
| --- | --- | --- | --- | --- | --- | --- | --- | --- | --- | --- | --- | --- | --- |
| Group R1 | 1 | F | Burkina Faso | 31 | Severe  bronchopneumonia | Pneumonia | Infections and infestations | ER | 6 | 426 | 14 | 3 | N |
|  | 2 | F | Ghana | 30 | Severe anemia | Anemia | Blood and lymphatic system disorders | HO | 5 | 812 | 1 | 3 | N |
|  |  |  |  | 30 | Cognegtive heart failure | Cardiac failure congestive | Cardiac disorder | HO | 5 | 812 | 1 | 3 | N |
|  |  |  |  | 30 | G6pd full defect | Glucose-6-  phosphate  dehydrogenase  deficiency | Congenital, familial  and genetic disorders | HO | 5 | 812 | 1 | 3 | N |
|  |  |  |  | 30 | Intravascular hemolysis | Intravascular hemolysis | Blood and lymphatic system disorders | HO | 5 | 812 | 1 | 3 | N |
|  |  |  |  | 30 | Malaria | Malaria | Infections and infestations | HO | 5 | 812 | 1 | 3 | N |
|  | 3 | M | Ghana | 2 | Failure to thrive | Failure to thrive | Metabolism and nutrition disorders | MD | 1 | 10 | 7 | 3 | N |
|  |  |  |  | 2 | Gastroenteritis | Gastroenteritis | Infections and infestations | MD | 1 | 10 | 7 | 3 | N |
|  |  |  |  | 2 | Malnutrition | Malnutrition | Metabolism and nutrition disorders | MD | 1 | 10 | 7 | 3 | N |
| Group R2 | 4 | M | Burkina Faso | 29 | Pharyngitis | Pharyngitis | Infections and infestations | MD | 7 | 365 | 4 | 2 | N |
|  | 5 | M | Burkina Faso | 8 | Fever | Pyrexia | General disorder and administration site conditions | MD | 6 | 137 | 1 | 3 | N |
|  | 6 | F | Burkina Faso | 39 | Septicemia | Sepsis | Infections and infestations | HO | 7 | 697 | 6 | 3 | N |
|  | 7 | M | Burkina Faso | 47 | Bacterial meningitis | Meningitis bacterial | Infections and infestations | HO | 7 | 911 | 13 | 3 | N |
|  |  |  |  | 47 | Salmonella septicemia | Salmonella sepsis | Infections and infestations | HO | 7 | 911 | 13 | 3 | N |
|  | 8 | M | Ghana | 9 | Accident | Accident | Injury, poisoning and procedural complications | ER | 6 | 141 | 1 | 3 | N |
| Group R3 | 9 | F | Burkina Faso | 28 | Septicemia | Sepsis | Infections and infestations | HO | 7 | 360 | 11 | 3 | N |
|  |  |  |  | 29 | Anemia | Anemia | Blood and lymphatic system disorders | HO | 7 | 364 | 7 | 3 | N |
|  |  |  |  | 29 | Streptococcal meningitis | Meningitis streptococcal | Infection and infestations | HO | 7 | 364 | 7 | 3 | N |
|  | 10 | F | Burkina Faso | 41 | Severe malaria | Malaria | Infections and infestations | MD | 7 | 694 | 4 | 3 | N |
|  | 11 | M | Burkina Faso | 3 | Bronchitis | Bronchitis | Infections and infestations | MD | 5 | 2 | 2 | 2 | N |
|  | 12 | M | Ghana | 6 | Bronchopneumonia | Pneumonia | Infections and infestations | HO | 6 | 76 | 16 | 3 | N |
|  |  |  |  | 7 | Hypertrophic cardiomyopathy | Hypertrophic cardiomyopathy | Congenital, familial and genetic disorders | HO | 6 | 89 | 3 | 3 | N |
| Group C1 | 13 | F | Burkina Faso | 10 | Anemia | Anemia | Blood and lymphatic system disorders | HO | 5 | 184 | 1 | 3 | N |
|  |  |  |  | 10 | Suspected bacterial sepsis | Bacterial sepsis | Infections and infestations | HO | 5 | 184 | 1 | 3 | N |
|  |  |  |  | 10 | Malaria | Malaria | Infections and infestations | HO | 5 | 184 | 1 | 3 | N |
|  | 14 | F | Burkina Faso | 10 | Suspected bleeding of digestive tract | Gastrointestinal hemorrhage | Gastrointestinal disorder | HO | 5 | 192 | 1 | 3 | N |
|  |  |  |  | 10 | Suspected septicemia | Sepsis | Infections and infestations | HO | 5 | 192 | 1 | 3 | N |
| Group C2 | 15 | F | Burkina Faso | 18 | Anemia | Anemia | Blood and lymphatic disorders | HO | 7 | 29 | 1 | 3 | N |
|  |  |  |  | 18 | Bronchiolitis | Bronchiolitis | Infections and infestations | HO | 7 | 27 | 3 | 3 | N |

Group R1 received RTS,S/AS01_E_ + (DTaP/Hib + tOPV + PHiD-CV), and HRV 2 weeks later, Group R2 received RTS,S/AS01_E_ + (DTaP/Hib + tOPV + HRV), and PHiD-CV 2 weeks later, Group R3 received RTS,S/AS01_E_ + (DTaP/Hib + tOPV), and (PHiD-CV + HRV) 2 weeks later, Group C1 received HepB + (DTaP/Hib + tOPV + PHiD-CV), and HRV 2 weeks later, Group C2 received HepB + (DTaP/Hib + tOPV + HRV), and PHiD-CV 2 weeks later.

AE, adverse event; Day of onset, latency following last dose administered (i.e. the time interval between the most recent dose of study treatment and the onset/start of the AE); Dose, number of doses of study treatment given prior the onset of the AE; DTaP/Hib, diphtheria-tetanus-acellular pertussis- *Haemophilus influenzae* type b; ER, emergency room; F, female; HepB, hepatitis B vaccine; HRV, human rotavirus vaccine; HO, hospitalization; Intensity, maximum severity of the event, i.e. mild (=1); moderate (=2) or severe (=3); M, male; MD, medical personnel; MED type, setting in which the event was observed and/or managed; N, No; RTS,S/AS01_E_, malaria vaccine; PHiD-CV, pneumococcal non-typeable *Haemophilus influenzae* protein D conjugate vaccine; tOPV, trivalent oral poliovirus vaccine.
