# Supplementary material for: Long-term immunogenicity and immune memory response to the hepatitis B antigen in the RTS,S/AS01E malaria vaccine in African children: a randomized trial
Source: Hum Vaccin Immunother. 2020 Jan 17;16(6):1464–70. doi: 10.1080/21645515.2019.1695457 (PMC7482624; doi:10.1080/21645515.2019.1695457)
Supplement: Supplemental Material [file KHVI_A_1695457_SM1032.zip › Supplementary Text.docx]

**Supplementary Text**

**Inclusion criteria of immunogenicity**

The according-to-protocol cohort for analysis of immunogenicity included all vaccinated and eligible participants who had received at least one dose of study vaccine according to their random assignment, who had not received a vaccine not specified or forbidden in the protocol, whose randomization was performed without failure and who received vaccine according to protocol for reason specified by the investigator, other than side, site and route or received a good replacement (not compatible with the vaccine regimen associated to the treatment number). Further inclusion criteria were that the participant met all eligibility criteria, did not receive any medication forbidden by the protocol, had not an underlying medical condition forbidden by the protocol, complied with vaccination and blood sampling schedule (including wrong and unknown dates), had no essential serological data missing/missing at all timepoints (if serology was present at any timepoint, even if not all, inclusion into ATP was allowed) and had received the complete vaccination course.
